# College Students Life Course Drawings And Their Descriptions

**DOI:** 10.1093/geroni/igab046.2294

**Published:** 2021-12-17

**Authors:** Amy Schuster, Katherina Terhune, Tina K Newsham, M Aaron Guest, Renee DuMont, Lauren Hackett, J R Patton, Kebafe Segosebe

**Affiliations:** 1 Clemson University, Clemson, South Carolina, United States; 2 Northern Kentucky University, Lexington, Kentucky, United States; 3 University of North Carolina Wilmington, University of North Carolina Wilmington, North Carolina, United States; 4 Arizona State University, Phoenix, Arizona, United States; 5 University of North Carolina Wilmington, Wilmington, North Carolina, United States

## Abstract

Drawing as a qualitative method has been employed to elicit views on aging. The subject matter of the drawings, without an explanation from participants, can be misinterpreted. Therefore, in this research, we explored college students’ drawings of the life course and the extent to which the content of these drawings corresponded to their written descriptions. A content analysis was performed on 524 college students’ life course drawings and their descriptions. Participants drew, on average, five life stages. The majority (75%) of the human beings represented were alone in each life stage. Twelve percent of the drawings were non-human representations of the life course (e.g., flower, tree). The majority of the images (85%) included in the drawings were not mentioned in the written descriptions, for example, hair changes (e.g., from long and straight to curly and short for women). Some physical characteristics (e.g., wrinkles [29%], hunched back [22%]) and some contexts (e.g., tombstones [37%], nursing home [100%]) were present in both the drawing and descriptions. Findings highlight which ideas associated with aging participants thought needed an explanation and which they might have seen as intrinsic to aging, warranting no explanation, emphasizing the importance of examining both drawn and written content when using drawing as a method in aging research. A more thorough and precise examination of the beliefs and perceptions of college students, who will serve as future professionals working with older adults, allows for the development of educational and engagement strategies that accurately target commonly held misperceptions regarding aging.

